# On Sterility, Treatment and Cure

**Published:** 1855-11

**Authors:** Wm. Cumming

**Affiliations:** Edinburgh—Vice President of the Edinburgh Obstetrical Society


### SELECTIONS.

*On Sterility depending on certain diseased states of the Lining Membrane of the Womb ; its treatment and cure.* By Wm. Cumming, M. D., F. R. C. P., Edinburgh—Vice President of the Edinburgh Obstetrical Society.

Cases of essential and incurable sterility depending on the female are extremely rare ; and it is not my purpose to refer to the cause of this form. But cases of removable sterility are very numerous ; and it may be interesting to detail some of the causes of it, the treatment, and the results.

I. One of these causes is a diseased state of a portion of the lining membrane of the uterus in cases of mal-position, the diseased part corresponding with the angle formed by the flexion of the womb on itself. According to my observation, the displacement of the womb most frequently accompanying this morbid condition of the mucous membrane, is anteversion, but other forms of displacement are not exempt from disease. I am now disposed to believe that no more displacement or contortion of the uterus will prevent impregnation, and that it is only when this is accompanied by a congested or ulcerated or otherwise diseased state of the lining membrane that it is a cause of sterility; and the reason of this seems to me to be, that the morbid part so flexed acts as a valve, which, while it allows a passage, painful or painless as may be, to the menstrual and muco-purulent discharges from within, refuses entrance to any fluid, such as the seminal, from without.

When these cases first came under my notice many years ago, being at that time inclined to attach more importance to mere displacement of the uterus than I now do, I attempted their cure by the means that were in use for the removal of the displacement. The chief means employed was the use of the intra-uterine pessary, on the supposition that there was no disease of the womb; but the success of this by no means corresponded to my expectations. It was evident that there was more than mere displacement, and that recourse must be had to other means; and having discovered in some (not certainly in all) a preternatural degree of tenderness and induration at the point of flexion, I was led to the conclusion that the mischief lay there, and that the treatment should be directed to that part. Accordingly, instead of inserting the pessary, I introduced bougies of different sizes till the constriction that existed at the angle of the displaced womb was removed, and followed this up by applying the solid nitrate of silver to the congested or otherwise diseased surface. The result of this was good. The painful menstruation was often removed, always relieved, a more free menstrual discharge followed, the intra-uterine leucorrhœa was by successive applications cured, and the patient in due time became pregnant. To this, of course, was added such treatment of the general system as seemed to be required.

Of this class, the following may be regarded as a not uncommon specimen :—

Mrs. Z———, married three years, had before marriage been more or less out of health at the menstrual period, but after that event, had her uterine symptoms much aggravated. She had for the two years previous to consulting me been treated by leeching, counter-irritation, &c., but without effect. She had also, on one occasion, had a bougie introduced within the os uteri, but the pain caused was so exquisite that the lady fainted, and the operation was not repeated. When she came under my care, I ascertained that there was very decided anteversion, great tenderness at the curve of the uterus—*i. e.*, at the part where it was anteverted on itself; and when I introduced a very small bougie for the purpose of examining the os internum, there was great tenderness and clearly some constriction. The leucorrhœal discharge was not very considerable, but it was *intra-uterine*, and irritated, and almost excoriated the vagina; and it was largest in quantity about midway between the menstrual periods.

It appeared to me that both the lady's illness and consequent sterility depended on the narrowness of the os internum, and probably also on the diseased state of the mucous membrane near it. There was no congestion either of the cervix or body of the womb, nor could I detect any other functional or organic derangement.

Having explained to the patient the nature of her case, and assured her that I could not undertake the treatment unless I was allowed to treat her by dilatation, to which, from her previous experience of the bougie, she had great objection, and having reduced considerably the tenderness of the diseased part by the inunction with belladonna ointment before introducing the bougie, I succeeded in dilating the os internum, and ultimately in applying the solid nitrate of silver. The effect of this was soon perceptible. She had much less painful menstruation, more of the discharge, and of a more natural character, and the examination of the affected part by the finger was much less painful. This was repeated from time to time for three months, when she left Edinburgh for her own home. About a year after, she returned, complaining that she was not yet cured, and proposed a consultation with another practitioner, who, after a careful digital examination, recommended incision of the os. To this she would not consent, and perhaps fortunately, for she was then nearly a month pregnant, and in due time was delivered of a very fine child, since which her health has been good, and her local symptoms have disappeared. I may add, that the last time she was in Edinburgh, —I mean at the time when she was a month pregnant,—the anteversion was as considerable as it had ever been; and except that she voided urine more frequently than natural, I am not aware that she suffered in any degree from the displacement.

This is a fair sample of a large number of cases, in which the treatment is neither severe nor protracted, and the result is very successful. The probability is, that the displacement and diseased condition of the mucous membrane have existed long before marriage, but have been aggravated by it. In such cases, so far as my experience goes, dilatation by bougies, and the application of the solid nitrate, effect a cure, are not liable to produce any dangerous or severe symptoms. In this respect the latter is much preferable to an injection of the solution—not a few disastrous results having followed the escape of the injection into the peritoneal cavity.

II. Another class of cases occurs similar to that now reported, but without a displacement of the womb. (By this term I mean flexion of the womb on itself, of so decided a character, that turn or twist it how you may with the uterine bougie it always reverts to the same mal-position, which is certainly not the case with many of what are called dislocations of the womb.)

The essential nature of this class appears to me to consist in marked constriction of the os internum, with ulceration of the lining membrane above the constriction and this ulcer often accompanied with induration of its base, and of part of the neighboring tissue. Whether the constriction precedes the ulceration or the reverse I do not pretend to say; but I have no doubt that ulcer increases the constriction, and that the removal of the former is essential to the cure. For this purpose I invariably dilate the os externum and internum and the cavity of the cervix, and apply the solid nitrate of silver very freely to the ulcerated or diseased surface, and with the best results, by which I mean removal of the local and general symptoms complained of by the patient, and, in time, of the sterility—I say in time, for in most cases impregnation does not occur for some time after the apparent cure.

I may mention that this constricted and ulcerated state of the lower part of the uterus produces two effects, which are calculated to mislead, and do very often mislead, practitioners. It induces a hypertrophied condition of the body, and a considerable enlargement of the cavity of the uterus: and till I was satisfied of this by a (comparatively) frequent occurrence of such cases I was inclined to regard, and did in reality often regard them as cases of hypertrophy, and so employed a treatment that not only failed of its anticipated effect, but weakened the patient, and greatly increased the local symptoms as well as reduced the general health. I am quite confident that no amount of depletion, either by leeches or scarification, and that no local application of ointment will remove the constriction and ulceration, though they may for a time relieve the congestion, heat, and irritation that generally accompany them, but which soon disappear without weakening treatment when their cause has been removed; and while I cannot help insisting that the repeated application of leeches in the treatment of uterine disease is very rarely necessary, I cannot help also declaring that I have known many cases in which the health of the patient has been seriously impaired, and life even compromised, by such treatment.

Any practitioner who has seen much of uterine disease may verify what I have said in regard to ulceration *within the womb*, by what he observes through the speculum in those cases where the ulceration is on the vaginal portion of the os externum. A patient with anxious, weary expression of countenance, and complaining of the ordinary local and general symptoms of leucorrhœa from ulceration, comes to consult us. On examination, we find the expected ulceration or abrasion, and some congestion. We apply the solid nitrate from time to time till the ulcer cicatrizes. We do not deplete nor mercurialize—in short, we do not weaken the patient. She recovers her health, her anxious expression vanishes, and in course of no long time she becomes pregnant. This, which we do see, as occurring at the *os externum*, occurs still more frequently at the *os internum* where we cannot see it, and precisely the same treatment is applicable the to one as to the other, with this addition, that before we can apply the nitrate of silver in the latter case we must dilate, and with this difference, that while the former or external species of ulceration almost invariably occurs in those who have had one or more children, the latter or internal, almost as invariably is found in virgins and those who have had none.

The following is one of many illustrating this form of the disease :—

Mrs. A——— has been married for nine months, when she had what was supposed to be a miscarriage; but from the menstruation, though for several months very scanty, having never been entirely suspended, and from a minute examination of the os and cervix uteri, I was quite satisfied that she had never been pregnant. When she first consulted me, she believed herself again at about the end of the third month of pregnancy; and as I was unwilling to incur the charge of having induced abortion, I contented myself with making a superficial examination, and waiting till time should put it beyond question one way or the other. She had however been regularly, though very scantily, menstruating, and though after a time she increased in size, and her mammæ (not the areola) enlarged and she had morning sickness, and many other of the signs and symptoms of pregnancy, I was quite confident that she was not pregnant. Still she and her friends deprecated interference, and she went on until she reached the (supposed) seventh month. At this time I was desirous to begin the treatment that I thought likely to remove both the disease and the sterility, and requested the opinion of a professional friend, who agreed with me as to the non-pregnancy. I then examined with a bougie, and found marked constriction at the os internum, and very acute pain, produced by the passage of the bougie—pain described as being similar to that produced by the extrusion of a small clot of blood during the menstrual period.

The leucorrhœa was intra-uterine, and in considerable quantity about midway between the two periods.

The treatment (local) consisted in dilatation of the passage as far as the cavity of the body of the womb; and in affecting this, I remarked what is, I believe very common in such cases, that after passing the constriction at the internal os the bougie reaches a cavity of much larger dimensions than natural, in which it can be moved about with freedom, and yet containing no polypus or tumor, and with its walls slightly increased in thickness, as if the effort to expel the clots at the menstrual period through the narrow neck had given rise to this form of hypertrophy with dilation, as is seen in the case of the heart. The dilatation was followed by the application of the solid nitrate of silver to the diseased part, and this was repeated till all tenderness was gone. Complete relief from pain during the menstrual period was the consequence, and utimately the patient become pregnant. She has since her delivery (which took place at the full time) enjoyed perfect health.

It were easy to detail many such cases, but they are all more or less alike.

III. Another cause of sterility is a diseased state of the lining membrane of the cavity of the uterus, not necessarily (though not unfrequently) accompanied by the constricted and ulcerated state of the cervix referred to in the preceding section.

The chief symptoms of this is the persistent continuance of uterine leucorrhœa in very considerable quantity, attended by the usual weakness, discomfort, irritability, and despondency, observed in most affections of the womb. The patient feels better at, and immediately before and after, the menstrual period, but feels all her ills heavy on her in the intermediate time.

In these cases the whole or greater part of the lining membrane of the womb is diseased. It is quite possible that the seminal fluid may pass into the womb, so as to come in contact with the ovum (impregnated), when it reaches the womb does not find a healthy point of contact and that therefore it passes through and perishes. In short, there is frequent impregnation, and as frequent destruction of the ovum. The object in view, therefore, is to restore a healthy state of the mucous membrane, and thus at the same time remove the disease and the sterility. The process of treatment is similar to that for the previous class of cases, with this difference, that the cavity of the body of the womb requires to be cauterized. This should be done at the end of the first week after the menstrual period, and repeated once a month till a healthy state and action are induced.

It is possible that the treatment of this class of cases may be conducted on different principles with success; but to me the plan mentioned seems the most simple, direct and successful, and it has this great recommendation, that it is quite as safe to apply the caustic to the inside as to the outside of the womb. I have said nothing of the general treatment; but though very important, there is nothing in it very different from what has been long and is every day pursued.

Mrs. M——— had been married for several years, and had enjoyed good health till her marriage. From that event she dated her complaints, all of which pointed to the uterine system. In this case the prominent symptoms was the uterine leucorrhœa between the menstrual periods, commencing a few days after the disappearance of the menstrual fluid, and continuing generally for ten days. She was comparatively well just before and after the menses, but when the white discharge appeared, she felt there was something wrong,—something that weakened and reduced her; and though she had undergone treatment of various kinds for it, the disease still persisted. On examination I could detect lateral displacement of the uterus, but no constriction. The body of the womb, however, was tender to the touch, and the bougie, when fully introduced, occasioned unusual pain. It appeared to me that the disease in this case lay in the cavity of the body; that the lining membrane was affected over a considerable surface; and that the treatment should consist in very free and repeated caustication of the whole mucous membrane. Having dilated the cervix with a sponge tent, I did so, and with a very gratifying result. The body of the womb became gradually less tender, the leucorrhœa discharge diminished, and lost its muco-purulent character; the vagina, which was tender from the acrid character of the discharge, became smooth and soft; the back lost its weakness; the general health became restored; and ultimately pregnancy supervened, with a favorable result.

In conclusion, I may add, that in connexion with this mode of treatment, there is a class of miscarriages in which this cauterization of the internal surface of the uterus is very successful—I mean, those in which an abortion has occurred between the second and third month, followed by general weakness, from which the patient does not recover, and the other uterine symptoms already detailed, and where a second pregnancy seems impossible. In these cases, examination with the uterine bougie generally communicates the feeling of its coming in contact with a rough, somewhat hard, uneven surface.—*London Lancet.*

